# Characteristics and Clinical Implications of Carbapenemase-Producing *Klebsiella pneumoniae* Colonization and Infection, Italy

**DOI:** 10.3201/eid2705.203662

**Published:** 2021-05

**Authors:** Marianna Rossi, Liliane Chatenoud, Floriana Gona, Isabella Sala, Giovanni Nattino, Alessia D'Antonio, Daniele Castelli, Teresa Itri, Paola Morelli, Sara Bigoni, Chiara Aldieri, Roberto Martegani, Paolo A. Grossi, Cecilia Del Curto, Stefania Piconi, Sara G. Rimoldi, Paola Brambilla, Paolo Bonfanti, Evelyn Van Hauwermeiren, Massimo Puoti, Gianni Gattuso, Chiara Cerri, Mario C. Raviglione, Daniela M. Cirillo, Alessandra Bandera, Andrea Gori, The KPC-Kp Study Group

**Affiliations:** S. Gerardo Hospital, Monza, Italy (M. Rossi, T. Itri, D. Castelli, P. Bonfanti);; Istituto di Ricerche Farmacologiche Mario Negri IRCCS, Milan, Italy (L. Chatenoud, I. Sala, G. Nattino, A. D’Antonio);; IRCCS San Raffaele Scientific Institute, Milan (F. Gona, C. Del Curto, D.M. Cirillo);; Fondazione IRCCS Ca’ Granda, Ospedale Maggiore Policlinico, Milan (T. Itri, A. Bandera, A. Gori);; Humanitas University, Milan (P. Morelli);; Papa Giovanni XXIII Hospital, Bergamo, Italy (S. Bigoni);; University of Milan San Paolo Hospital, Milan (C. Aldieri);; Busto Arsizio Hospital, Busto Arsizio, Italy (R. Martegani);; University of Insubria, ASST Sette Laghi-Varese, Italy (P.A. Grossi);; ASST Fatebenefratelli Sacco, Milan (S. Piconi, S.G. Rimoldi);; Clinic of Infectious Diseases of Istituti Ospedalieri of Cremona, Cremona, Italy (P. Brambilla);; University of Milan-Bicocca, Milan (P. Bonfanti);; University of Brescia and ASST Spedali Civili, Brescia, Italy (E. Van Hauwermeiren);; ASST Grande Ospedale Metropolitano Niguarda, Milan (M. Puoti);; Carlo Poma Hospital, Mantova, Italy (G. Gattuso); Hospital of Lodi, Lodi, Italy (C. Cerri);; University of Milan, Milan (M.C. Raviglione, A. Bandera, A. Gori)

**Keywords:** *Klebsiella pneumoniae*, Carbapenem resistance, KPC-*Kp*, *Enterobacteriaceae*, CRE, antimicrobial resistance, mortality rates, bacteria, healthcare-associated infections, Italy

## Abstract

*Klebsiella pneumoniae* carbapenemase–producing *K. pneumoniae* (KPC*-Kp*) has been endemic in Italy since 2013. In a multicenter cohort study, we investigated various aspects of KPC*-Kp* among patients, including 15-day mortality rates and delays in adequate therapy. Most (77%) KPC*-Kp* strains were sequence type (ST) ST512 or ST307. During 2017, KPC-*Kp* prevalence was 3.26 cases/1,000 hospitalized patients. Cumulative incidence of KPC-*Kp* acquired >48 hours after hospital admission was 0.68% but varied widely between centers. Among patients with mild infections and noninfected colonized patients, 15-day mortality rates were comparable, but rates were much higher among patients with severe infections. Delays of >4 days in receiving adequate therapy more frequently occurred among patients with mild infections than those with severe infections, and delays were less common for patients with known previous KPC*-Kp* colonization. Italy urgently needs a concerted surveillance system to control the spread of KPC-*Kp*.

## Abstract 2

We found 15-day mortality rates were higher for patients with severe infections than for those with mild infections or colonization.

The global emergence and spread of carbapenem-resistant *Enterobacteriaceae* (CRE) pose a major health threat, causing severe illness and high healthcare costs (*1*). Infections caused by CRE also are associated with high mortality rates because extensive resistance to so-called last-line antimicrobial drugs, such as carbapenems, limit the treatment options (*2*–*5*). Only a few antimicrobial drugs, such as colistin, fosfomycin, tigecycline, and ceftazidime/avibactam, are effective against CRE. Moreover, the remaining therapeutic options often have high toxicity profiles, and rates of resistance to these antimicrobial drugs already are increasing (*6*).

In a 2014 study conducted by the European Survey of Carbapenemase-Producing Enterobacteriaceae (EuSCAPE) Working Group, 455 sentinel hospitals in 36 countries submitted clinical isolates (*7*). Among the 2,703 isolates submitted, 2,301 (85%) were *Klebsiella pneumoniae* and 402 (15%) were *Escherichia coli*, including samples identified as carbapenemase producers among 850 (37%) *K.* *pneumoniae* and 77 (19%) *E. coli* isolates. Identified carbapenemase-producers included 4 gene families: *K. pneumoniae* carbapenemase (KPC), New Delhi metallo-β-lactamase, oxacillinase 48-like, and Verona integron-encoded metallo-β-lactamase (*7*). Positive clinical specimens were found in 1.3 patients/10,000 hospital admissions, but prevalence differed greatly between countries and the highest rates were registered in countries in the Mediterranean and Balkan regions (*7*). Among these countries, Italy, Greece, and Romania reported the highest percentages of carbapenem resistance. In addition, CRE rates increased from 15% in 2010 to 36% in 2016 (*8*–*10*), and CRE became endemic in Greece in 2010 and Italy in 2013 (*11*). Nevertheless, currently published information is too scant to define the complete picture of KPC *K.* *pneumoniae* (KPC-*Kp*) epidemiology in both clinical isolates and surveillance screening samples (*12*).

In this context, we set up a network of 15 hospitals in Lombardy, the most populous region in Italy, and established a cohort of patients affected by KCP-*Kp*. The overarching goal of the KPC-*Kp* Study Group was to identify the challenges of controlling the spread of the bacterium. We describe KPC-*Kp* epidemiology, treatment, and in-hospital mortality rates, along with molecular characterization of KPC-*Kp* strains in colonized and infected inpatients.

## Methods

### Study Design, Setting, and Patients

**Figure 1:**
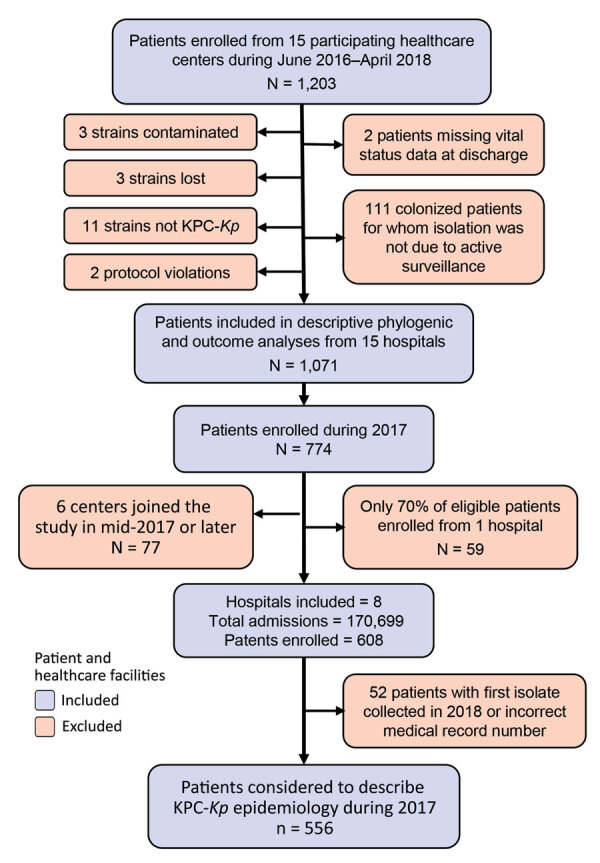
Flow chart of network of healthcare centers participating in a study of *Klebsiella pneumoniae*–carbapenemase producing *K. pneumoniae* (KPC-*Kp*), Italy, 2016–2018. The KPC-*Kp* network included 15 hospitals. Patients were included when KPC-*Kp* was diagnosed and excluded for various reasons. Hospitals were included when they submitted KPC-*Kp*–confirmed isolates and excluded from analysis when had no confirmed patients or did not enroll all confirmed patients. KPC-*Kp*, *Klebsiella pneumoniae*–carbapenemase producing *K. pneumoniae*.

We conducted a multicenter cohort study during June 2016–April 2018, which included 15 hospitals in Lombardy (Figure 1). We asked each enrolled hospital to include data on all consecutively hospitalized adult patients who had >1 positive KPC-*Kp* isolate during their hospital stay. For patients hospitalized multiple times during the study period, we only considered the first hospitalization. For centers including patients during 2017, the year for which we had a full 12 months of data, we retrieved the administrative datasets of all admitted patients (Figure 1). We merged these data with those available in the KPC-*Kp* patient cohort database and used the combined dataset to describe KPC-*Kp* epidemiology in the hospitalized population. 

The study protocol was first approved by the Research Ethics Committee of the coordinating center, Ospedale San Gerardo (Monza, Italy). Informed consent requirement was waived due to the study’s observational, noninterventional design. The study protocol was subsequently approved by the ethics committees of the 14 other participating centers. In accordance with local ethics committee requirements, 3 centers did not waive informed consent. Because this was an observational study, treatment for KPC-*Kp* infections was at the discretion of the attending physicians and no change to the center-specific surveillance protocol was required.

In all centers, intensive care unit (ICU) patients were tested for CRE at admission and weekly through rectal swab specimens or other surveillance cultures. The same protocol was applied heterogeneously in hospital wards in which patients are considered to be at higher risk of acquiring CRE, such as hematology, solid organ transplant, and geriatric units (Appendix Table 1). For the other wards, most centers performed surveillance rectal swab specimens at admission on the basis of major risk factors for CRE, such as previous CRE colonization, previous hospitalization during the 12 months before inclusion, or both. Of note, only 3 of the 15 participating centers, B, C, and I (Appendix Table 1), combined the 2 surveillance strategies described for specific wards and patients at higher risk of acquiring CRE.

### Patient Classification

Patients were classified according to the most clinically relevant KPC-*Kp* isolate collected from them between hospital admission and discharge. Thus, for patients whose first isolate was attributable to colonization and a subsequent isolate was attributed to an infection, only the second isolate was considered. We used US Centers for Disease Control and Prevention criteria (*13*) to define diagnosed infection and diagnosis was confirmed by an infectious disease specialist. Infections were classified as KPC-*Kp* bacteremia when a blood culture was positive for a KPC-*Kp* strain with or without KPC-*Kp*–positive cultures from >1 other site and the patient had clinical signs of systemic inflammatory response syndrome requiring antimicrobial drug treatment. We defined nonbacteremic KPC-*Kp* infections by documented recovery of a KPC-*Kp* isolate from nonblood cultures, such as intra-abdominal wounds, urine, or bronchoalveolar lavage fluid; absence of KPC-*Kp*–positive blood culture during the index hospitalization; and clinical signs of infection.

In line with other studies (*14*), we classified KPC-*Kp* cases according to infection severity. We classified cases of KPC-*Kp* bloodstream or lower respiratory tract infections, and clinical presentation of septic shock, regardless of infection site, as severe infections. We classified infections from the urinary tract, surgical wounds, or other sites without septic shock as mild infections. We classified all cases identified through active surveillance as colonized when >1 culture sample grew KPC-*Kp* but the patient did not develop KPC-*Kp* infection during hospitalization.

### Data Collection

For patients included in the KPC-*Kp* cohort, data were entered into the web-based case form after pseudonymization of personal data. Data were collected on demographic characteristics, medical history, underlying diseases, previous hospitalization, previous KPC-*Kp* infection, surgery <30 days before KPC-*Kp* isolation, invasive procedures <72 hours before KPC-*Kp* isolation, antimicrobial drug therapy <30 days before KPC-*Kp* isolation, dates of admission to hospital, and ward of isolation. Date of hospital discharge and patient status at discharge also were collected. The date and ward where the patient was hospitalized when KPC-*Kp* was isolated, the source of isolation, and resistance spectrum also were collected and entered into the web-based case record form. Antimicrobial treatment, including empirical treatment and post-antibiogram treatment regimen, were recorded. Empirical treatment was defined as adequate when it included >1 antimicrobial drug with in vitro activity against the KPC-*Kp* isolate. Data were collected in a web-based case report form.

For enrolled centers submitting patient data during 2017, we retrieved the clinical record datasets of all admitted patients after pseudonymization of personal information. To verify centers included all eligible patients, we retrieved the total number of patients with >1 KPC-*Kp*–positive isolate registered in the microbiology laboratory of each center and compared that with the total number of patients included in the cohort (Appendix).

### Microbiology and Genomic Analysis 

The clinical microbiology laboratory of each of the 15 participating centers performed isolate identification and routine antimicrobial susceptibility testing (Appendix). CRE was defined by using Clinical and Laboratory Standard Institute guidelines (*15*). All bacterial strains were sent to a central microbiological laboratory at Ospedale San Raffaele for whole-genome sequencing (Appendix).

### Statistical Analysis

We estimated the prevalence of KPC-*Kp* in hospitalized patients in the region of Lombardy during 2017, the cumulative incidence of acquired KPC-*Kp* infections among hospitalized patients, and the cumulative incidence of acquired KPC*-Kp* infections occurring >48 hours after hospital admission among hospitalized patients in the same region. We calculated and reported crude estimates for all centers and estimates standardized by age and ward of isolation (Appendix).

To study the role of KPC-*Kp* infection severity on 15-day mortality rates, we considered a multivariable Cox proportional hazard model and the related hazard ratio (HR) estimates and adjusted by center for a random effect and number of days from hospitalization to KPC-*Kp* isolation. Colonized patients frequently have shorter hospital stays than infected patients. Because a shorter discharge time could affect our results, we performed a sensitivity analysis in which we excluded early-discharge patients. We performed a subgroup analysis to quantify excess mortality hazard due to septic shock among patients with bloodstream infections (Appendix).

We used multivariable mixed logistic regression models and accounted for clustering at the center level to evaluate the association between patient characteristics and delayed or inadequate empirical therapy, which we considered as outcome variables. We adjusted the models for age and type of KPC-*Kp* infection.

## Results

### Center Characteristics

Among all centers, the median number of annual admissions was 27,600 (interquartile range [IQR] 18,287–40,000). Among 15 enrolled centers, 9 (60%) maintained enrollment over 12 consecutive months; centers had a mean enrollment duration of 13.8 months (Appendix Figure 1).

### Patient Baseline Characteristics

Among 1,203 consecutive KPC-*Kp*–positive hospitalized patients found during study, 89.0% (1,071) were considered in the analyses and 11% (132) were excluded for various reasons (Figure 1). The median age among patients was 72 (IQR 61–80) years, 65% were male, and 35% were female; KPC-*Kp* was isolated from 275 (25.7%) ICU patients (Table 1). Among patients in the study cohort, >90% had >1 underlying condition, 40% of whom had congestive heart failure, peripheral vascular disease, or chronic renal failure. Severe infections were diagnosed in 221 (20%) patients and mild infections in 109 (10%) patients. Colonized patients (n = 741, 69.2%) had a median of 6 days between hospitalization and KPC-*Kp* isolation, which was much lower than for patients with severe (median 12 days) or mild (median 11 days) infections. Bloodstream infections accounted for 54% of all infections, and rectal swab samples accounted for 67% of all colonizations (Appendix Figure 2).

**Table 1:** Characteristics of patients identified in multicenter surveillance for *Klebsiella pneumoniae* carbapenemase-producing *Klebsiella pneumoniae*, Italy*

Characteristics	KPC-*Kp* patients, n = 1,071
Sex	
M	694 (64.8)
F	377 (35.2)
Median age (IQR)	72 (61–80)
Ward of isolation	
Intensive care unit	275 (25.7)
Infectious diseases	81 (7.6)
Surgery	149 (13.9)
Geriatrics	47 (4.4)
Oncology	34 (3.2)
Hematology	42 (3.9)
Other medical wards	443 (41.4)
KPC-*Kp* colonization in previous 12 mo	333 (31.1)
Hospitalization in previous 12 mo	865 (80.8)
Antimicrobial therapy in the 30 d before hospitalization	782 (73.0)
Major surgery in the previous 30 d	262 (24.4)
Underlying conditions†	989 (92.3)
Congestive heart failure	192 (17.9)
Peripheral vascular disease	197 (18.4)
Cerebrovascular disease	205 (19.1)
Chronic lung disease	202 (18.9)
Chronic renal failure	304 (28.4)
Cancer	244 (22.8)
Diabetes	163 (15.2)
Charlson index, median (IQR)	6 (4–8)
Central venous catheter at isolation	414 (38.7)
Urinary catheter at isolation	562 (52.5)
Immunosuppressive therapy	209 (19.5)
Days of hospitalization, median (IQR)	25 (14–45)
KPC-*Kp* acquisition characteristics‡	
Severe infection	221 (20.6)
Mild infection	109 (10.2)
Colonization_sur_	741 (69.2)
Median time from hospitalization to isolation of strain, d (IQR)‡	
Severe infection	12 (2–22)
Mild infection	11 (2–25)
Colonization_sur_	6 (1–17)
Median time from strain isolation to discharge or death, d (IQR)‡	
Severe infection	18 (9–35)
Mild infection	20 (12–35)
Colonization_sur_	13 (6–22)

*Values are no. (%) except as indicated. IQR, interquartile range; KPC-*Kp*, *Klebsiella pneumoniae* carbapenemase-producing *Klebsiella pneumoniae*. †Underlying conditions and devices are listed when present in >10% of patients. ‡Severe infection included bloodstream or lower respiratory tract infection plus septic shock from other sites; Mild infection included infections from other sites; and colonized_sur_ patients were identified through surveillance protocols.

### Distribution, Phylogeny, and Resistance Mechanisms of KPC-*Kp* Clones

**Figure 2:**
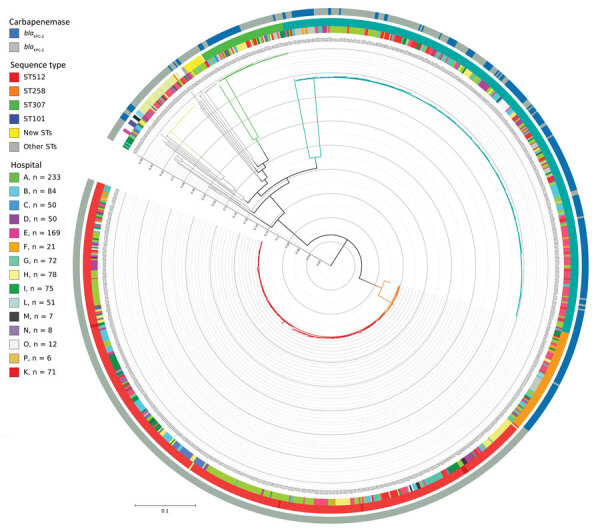
Phylogenetic tree of 989 *Klebsiella pneumoniae* genomes isolated at hospitals participating in the KPC-producing *K. pneumoniae* (KPC-*Kp*) study, Italy. The key shows the number of isolates included in the study provided by each center; 2 samples (1 from each from hospitals A and I) were excluded because the total quality of the assemblies was not sufficient to have high confidence in the SNPs called through all the genome (total coverage <30). Inner circle shows the KPC-*Kp* mechanism identified; middle circle shows hospitals from which strains were isolated; and the outer circle the shows identified STs. The whole genome core single-nucleotide polymorphisms (SNPs) were extracted from the 989 *K. pneumoniae* genome assemblies by using kSNP3.0 (https://sourceforge.net/projects/ksnp). Parametric maximum-likelihood estimation (general time-reversible plus gamma distribution plus invariable sites) analysis with 1,000 bootstrap estimates was used to infer the phylogeny. We used IQ-TREE (http://www.iqtree.org) to generate the tree and iTOL (https://itol.embl.de) to draw the tree. Major STs are represented by branch colors; ST512 and ST307 were the predominant STs. Major branches have bootstrap values >0.75 for branch support. Scale bar indicates nucleotide substitutions per site. KPC, *Klebsiella pneumoniae*–carbapenemase; ST, sequence type.

Among the 1,071 patient strains isolated, 82 were from colonized patients included at the end of April 2018; these samples did not arrive at the central laboratory in time for genotyping. Of the 989 strains analyzed, 32 different sequence types (STs) were identified. The most numerous clones were ST512 in 45% (441), ST307 in 33% (326), ST258 in 7% (71), and ST101 in 6% (57) of isolates (Appendix Figure 3). We identified 2 KPC variants, KPC-2 and KPC-3, in 68% of isolates. KPC-2 was absent in ST512 but predominant in ST307 and ST258. Core-genome, single-nucleotide polymorphism (SNP) analysis revealed that ST512 was scattered across all centers, but ST307 was represented in smaller, more localized clusters (Figure 2; Appendix Table 3).

### Epidemiology of KPC-*Kp*

During 2017, the estimated prevalence of KPC-*Kp* among hospitalized patients in the Lombardy region was 3.26 (95% CI 2.99–3.54) per 1,000 admissions. In the same region, the overall cumulative incidence of KPC-*Kp* infections was 1.00‰ (95% CI 0.86‰–1.16‰) and the incidence of acquired infections occurring >48 hours after hospital admission was 0.68‰ (95% CI 0.56‰–0.82‰). The proportion of patients infected at admission, considered imported infections, was ≈30% in most centers. We observed marked differences across centers even after standardization by age and ward of isolation, with values ranging from 1.62‰ (95% CI 1.07‰–2.18‰) in center A to 0.21‰ (95% CI 0.02‰–0.40‰) in center B (Appendix Figure 3).

### Patient Outcomes

In-hospital death from all causes was 34% (95% CI 29.2%–39.6%) among KPC-*Kp*–infected patients and 21% (95% CI 17.7%–27.6%) among colonized patients. No differences emerged when we stratified for carbapenem-resistance mechanisms and the most prevalent clones (Appendix Table 4).

Mortality hazards (considering the first 15 days after KPC-*Kp* isolation) were much higher for patients with severe infection than for colonized patients, even after controlling for center, time between hospitalization and isolation, age, ward of isolation, and Charlson index (adjusted HR [aHR] = 1.93, 95% CI 1.40–2.66) (Table 2). In contrast, no excess mortality hazard was noted for patients with mild infections (aHR = 0.75, 95% CI 0.42–1.34) compared with colonized patients. When we analyzed the subgroup of patients with bloodstream infections, we found clinical manifestation of septic shock more than doubled the risk for death (HR = 2.71, 95% CI 1.46–5.02). We found comparable results when we excluded from the analysis 343 patients discharged alive before day 15 (data not shown).

**Table 2:** In-hospital death within 15 days of KPC-*Kp* isolation in a cohort of infected patients and subgroup of patients with bloodstream infections, Italy*

KPC-*Kp* infections	No.	Died, no. (%)	HR (95% CI)†	p value	HR (95% CI)‡	p value
All patients	1,039	174 (16.7)	NA	NA	NA	NA
Severity of infection§						
Colonized	712	100 (14.0)	Referent	NA	Referent	NA
Mild	109	13 (11.9)	0.71 (0.40–1.27)	0.247	0.75 (0.42–1.34)	0.328
Severe	218	61 (28.0)	1.84 (1.34–2.54)	0.0002	1.93 (1.40–2.66)	<0.0001
Bloodstream infections	176	45 (25.6)	NA	NA	NA	NA
Septic shock at admission						
N	132	25 (18.9)	Referent	NA	Referent	NA
Y	44	20 (45.5)	2.72 (1.50–4.90)	0.0009	2.71 (1.46–5.02)	0.002

*All patients are stratified for severity of infection; the subgroup of patients with bloodstream infection is stratified for septic shock. KPC-*Kp, Klebsiella pneumoniae* carbapenemase-producing *Klebsiella pneumoniae*; NA, not applicable.  †Hazard ratio (HR) estimates are from multivariable Cox proportional hazard models, adjusting for center (random effect) and days elapsing from hospitalization to KPC-*Kp* isolation. ‡Multivariate Cox mixed effects model adjusting for center (random effect) and days elapsing from hospitalization to KPC-*Kp* isolation, age, Charlson Index, and whether or not isolates were collected when patient was in the intensive care unit. §Patients discharged or deceased on the day of KPC-*Kp* isolation were excluded from analyses; 20 patients were discharged, 9 colonized patients died, and 3 colonized patients had severe infections.

### Antimicrobial Drug Treatment

On the basis of susceptibility test results, we found that 54% (159/297) of patients infected with KPC-*Kp* received adequate empirical therapy (Appendix Table 5). Empirical treatment was most frequently adequate in patients with KPC-*Kp* colonization during the previous 12 months and in patients with severe infection (Appendix Table 5).

Fewer treatment delays (<4 days, which is considered the maximum acceptable waiting time to receive appropriate antimicrobial treatment) were reported for patients with severe KPC-*Kp* infection than patients with mild infections (Table 3). Patients reporting KPC-*Kp* colonization during the previous 12 months more frequently received prompt adequate therapy (p<0.001).

**Table 3:** Association between delay in receiving adequate antimicrobial therapy after KPC-*Kp* isolation and selected patient characteristics, Italy*

Characteristics	Delay from KPC-*Kp* isolation to adequate antimicrobial therapy		χ^2^ p value	p value†
	<4 d	>4 d		
All	190 (63.9)	107 (36.0)	NA	NA
Age, median (IQR)	68.5 (62–78)	74 (63–81)	0.151	0.285
Charlson Index, median (IQR)	5.0 (4–8)	6.0 (4–8)	0.615	0.439
Intensive care unit admission				
Y	41 (63.1)	24 (36.9)	0.865	0.354
N	149 (64.2)	83 (35.8)		
Previous KPC-*Kp* colonization during the current hospitalization				
Y	46 (74.2)	16 (25.8)	0.060	0.118
N	144 (61.3)	91 (38.7)		
KPC-*Kp* colonization in the previous 12 mo				
Y	104 (77.0)	31 (23.0)	<0.001	<0.001
N	86 (53.2)	75 (46.8)		
Hospitalization in the previous 12 mo				
Y	149 (64.5)	82 (35.5)	0.832	0.779
N	41 (63.1)	24 (36.9)		
Antimicrobial therapy in the 30 d before hospitalization				
Y	145 (64.0)	84 (36.0)	0.564	0.627
N	45 (67.2)	22 (32.8)		
Major surgery‡				
Y	48 (53.9)	41 (46.1)	0.018	0.008
N	142 (74.7)	66 (31.7)		
KPC-*Kp* infection severity§				
Severe	139 (71.5)	55 (28.3)	0.0002	<0.001
Mild	52 (50.0)	52 (50.0)		

*Values are no. (%) except as indicated. Delay determined according to infected patients’ resistance profiles; 33 patients were excluded: 17 had follow-up <3 days after isolation and 16 had no data on empirical therapies. IQR, interquartile range; KPC-*Kp*, *Klebsiella pneumoniae-*carbapenemase producing *Klebsiella pneumoniae*; NA, not applicable. †Obtained from multivariable mixed logistic model adjusted by center, as random effect; age; and type of KPC-*Kp* infection, when appropriate. ‡Major surgery includes *any invasive operative procedure in which a more extensive resection is performed,* *including a body cavity is entered*, *organs are removed, or normal anatomy is altered.*  §Severe infection included bloodstream or lower respiratory tract infection plus septic shock from other sites; Mild infection included infections from other sites; and colonized patients were identified through surveillance protocols.

Among the 282 KPC-*Kp*–infected patients treated for their infections, 62 (22%) received an in vitro active drug plus carbapenem, but 29 (10%) patients received gentamicin, fosfomycin, or tigecycline monotherapy. The most common drug combination was colistin plus tigecycline plus carbapenem, which most frequently was administered to patients with severe infections. Ceftazidime/avibactam became available in Italy in February 2018, and 26/39 (66%) infected patients included after that date received it: 19/24 (79%) in the severe infection group and 7/15 (47%) in mild infection group (Appendix Table 6). 

## Discussion

This study provides a detailed picture of KPC-*Kp* burden in an endemic setting and shows that KPC-*Kp* poses a major challenge for Italy’s healthcare system. We estimated that 1 of every 1,000 patients admitted to participating hospitals during 2017 had a positive KPC-*Kp* specimen during hospitalization, which is ≈10 times the estimated number of CRE infections in Europe (1.3/10,000 hospitalizations) (*7*). This high rate is at least partly compatible with the heterogeneity in the surveillance protocols adopted by hospitals. Another factor contributing to the high rate of KPC-*Kp* could be the older age of the patient population, most of whom were men >65 years of age. In 2017, the median age of the adult population in Lombardy was 50 years, but the median age for the 170,699 adult patients in our study was 66 years, and 27% were >77 years of age. Of note, the considerable proportion of imported KPC-*Kp* infections, ≈30%, for most centers, suggests that active surveillance might need to be extended to post-acute care, long-term care, or rehabilitation facilities to control the spread of KPC-*Kp*. As highlighted by a recent report from the European Centre for Disease Prevention and Control (*16*), standardized actions for CRE containment in Italy must be driven by comprehensive coordinated responses implemented nationally rather than current practice of delegating responsibilities to the regional or hospital level.

In our setting, the KPC-*Kp* epidemic appears to be driven by the expansion of 3 major *K. pneumoniae* clonal lineages, specifically ST307, ST101, and ST258/ST512. These epidemic clones have been associated with outbreaks and are reported to have an increased capacity to acquire drug resistance (*17*–*19*). Clone ST512 was widely distributed across the centers in our study, confirming its spread in Italy (*20*). We noted clone ST307 in smaller, scattered clusters but did not note differences in infection severity or death between clones.

We examined the KPC-*Kp*–associated mortality rate and noted it was highest among patients with severe infections, particularly bloodstream infections with septic shock, which is consistent with previous research (*21*–*25*). We found no excess risk for death among patients with mild infection. KPC-*Kp* often is found in vulnerable hospital populations at high risk for illness and death (*21*,*26*). To estimate the effect of KPC-*Kp* infection on hospital mortality rates, we compared patients with severe and mild infections with colonized patients. Colonized patients who did not have infectious events during hospitalization represented the best available control group because they were hospitalized in the same hospitals at the same time as KPC-*Kp* infected cases and are known to have similar clinical characteristics and underlying conditions (*27*). 

Regarding therapeutic approaches, we found the initial empirical selection of antimicrobial drug treatment was more frequently adequate in patients with a known previous KPC-*Kp* colonization. This result is in line with other published studies reporting that for patients with no history of previous colonization, adequate antimicrobial treatment can only be started once the susceptibility profile has been received, and this delay might lead to unfavorable outcomes (*28*–*31*). Thus, in geographic regions with high CRE prevalence, extending rectal swab specimen surveillance to a broader at-risk hospital population is crucial to reduce time to adequate antimicrobial therapy and, ultimately, to improve patients’ outcomes. As previously observed (*4*,*29*), a combination of >2 active agents have been prescribed predominantly in patients with severe infections and at higher risk for death. Of note, we observed a substantial use of colistin despite its unknown efficacy and poor safety profile (mainly related to renal failure), as documented in other studies (*32*–*34*). In addition, ceftazidime/avibactam use has increased since 2018, when it became available for routine clinical use in Italy. However, the use of ceftazidime/avibactam in nonbacteremic infections should be discouraged to reduce chances of acquired in vitro resistance (*35*–*37*). The wide variety of therapeutic regimens, >30 combinations reported in our centers, confirms the need for multicenter randomized trials to identify the most effective combination and dosage of antimicrobial agents.

The major strengths of our study are the size of the sample and the representation of KPC-*Kp* patients included with homogeneous methodology through an independent network of Lombardy hospitals of different size. The results reveal the multifaceted reality of KPC-*Kp* infection in clinical settings.

The first limitation of our study is that we focused on the most clinically relevant episode for each patient. Therefore, patients who had a colonization followed by an infection were considered and classified according to this second more severe event only. However, in our setting, this subgroup included only 8% of the colonized patients. Second, we limited our attention to KPC-*Kp* strains, ignoring *E. coli* and other carbapenemase, such as oxacillinase 48-like and New Delhi metallo-β-lactamase. Nevertheless, the estimated ratio of *K. pneumoniae* to *E. coli* was 11:1 in Italy (*16*), and KPC is the only endemic mechanism demonstrating carbapenem resistance (*9*). Third, despite the inclusion of a large number of infected patients, the multitude of treatment patterns prevented reliable exploration of effects of treatment on clinical outcomes, but the description of this heterogeneity remains one of the findings of this study. Finally, we focused on overall rather than disease-specific mortality rates because we aimed to give a global picture of KPC-*Kp* burden in the Lombardy region. Cause-specific mortality analysis would have required detailed information on the procedures performed before the events occurring during hospitalization, which was beyond the scope of this study.

In conclusion, our study describes KPC-*Kp* in a single region of Italy where KPC-*Kp* has been endemic since 2013. The KPC-*Kp* epidemic appears to be driven by the expansion of only 3 major clonal lineages. Therefore, the wide heterogeneity in the proportion and incidence of KPC-*Kp* infections are presumably largely influenced by surveillance protocols and hospital policies. Consequently, to reverse this trend, Italy needs a strengthened collaborative surveillance system that includes regional plans and strong, centrally coordinated activities at the national level. Furthermore, the wide range of treatments adopted by healthcare facilities in this study highlights the urgent need to accompany the surveillance system with a concerted, aggressive, and prompt antimicrobial stewardship plan.

Appendix Additional information on characteristics and clinical implications of *Klebsiella pneumoniae-*carbapenemase producing *Klebsiella pneumoniae* in Italy.
