# Supplementary material for: Characteristics and Clinical Implications of Carbapenemase-Producing Klebsiella pneumoniae Colonization and Infection, Italy
Source: Emerg Infect Dis. 2021 May;27(5):1416–26. doi: 10.3201/eid2705.203662 (PMC8084501; doi:10.3201/eid2705.203662)
Supplement: Appendix — Additional information on characteristics and clinical implications of Klebsiella pneumoniae-carbapenemase producing Klebsiella pneumoniae in Italy. [file 20-3662-Techapp-s1.pdf]

Article DOI: https://doi.org/10.3201/eid2705.203662

Characteristics and Clinical Implications of Carbapenemase-Producing *Klebsiella pneumoniae* Colonization and Infection, Italy

Appendix

Extended Methods

Study Design, Setting and Patients

Center Characteristics

The study was conducted in 15 healthcare centers in Lombardy, the most populous region in Italy with 10,000,000 inhabitants (16.5% of the Italian population). These represent all regional centers with both a microbiology and an infectious diseases unit. The centers are distributed across the regional territory, providing extensive coverage. Among 15 participating centers, 8 are large tertiary care institutions with >25,000 admissions per year, offering a full range of clinical and surgical services (Appendix Table 1). All hospitals had a general medicine unit, various branches of surgery and ≥1 intensive care unit (ICU). Our study setting included a considerable proportion of immunosuppressed patients: 13 centers had a hematology unit and 8 a solid organ transplantation unit. In addition, 4 centers had a geriatric ward (Appendix Table 1).

In most centers, patients were empirically treated with currently standard doses of drugs with known gram-negative activity, either alone or with other antimicrobial drugs. Because the study is observational, empirical treatment regimens were at the discretion of the treating physician, in most cases in agreement with the infectious disease consultant, without the aid of a predefined protocol. Moreover, in some centers the policy of empirical therapy was based on 2 factors: the severity of the infection and the presence of risk factors for carbapenem-resistant *Enterobacteriaceae* (CRE).

Patient Infection Classification

When a single patient had multiple KPC-*Kp* infections, the most clinically relevant infection was considered in the analysis. For example, if a patient experienced a urinary tract infection followed by a bloodstream infection, only the bloodstream infection and related isolate were considered.

Personal Data Pseudonymization Process

The patient's name and date of birth were pseudonymized automatically with the generation of a patient identification number saved in the database. Only center staff could visualize personal data saved in a separate file.

Definition of Acquired Infections Adopted in All Centers

Patients with acquired infections were those with an infection arising >48 hours of admission (*1*). For infectious events, clinical presentation of septic shock, defined as sepsis with organ dysfunction and persistent hypotension despite volume replacement (*2*); chronic renal failure; antimicrobial treatment including empirical treatment, which was considered adequate when it included ≥1 drug with in vitro activity against the KPC-*Kp* isolate; and post-antibiogram treatment regimen were recorded.

Microbiology and Genomic Analysis of Strains

During the study period, the laboratory of each of the 15 participating centers collected consecutive, nonreplicate, clinical isolates of KPC-*Kp* from any site of infection or colonization of patients enrolled in the study.

Isolates were identified with the Vitek 2 system (bioMérieux, https://www.biomerieux.com) or matrix-assisted laser desorption/ionization-time-of-flight mass spectrometry by MALDI Biotyper (Bruker Daltonics GmbH, https://www.bruker.com) or Vitek-MS (bioMérieux). Each hospital conducted antimicrobial susceptibility testing via automated systems and according to standard protocols. Eleven centers used the Vitek 2 system (bioMérieux); 3 centers used Phoenix (Becton Dickinson, https://www.bd.com); 1 center used MicroScan Walkaway (Siemens, https://www.siemens.com). One center confirmed all MICs by broth microdilution method, whereas others confirmed only selected antimicrobial MICs by broth microdilution or E-test. Results were interpreted in accordance with the European Committee on Antimicrobial Susceptibility Testing (EUCAST) clinical breakpoints (*3*).

All centers performed phenotypic detection of carbapenemase types by combination disk/MHT according to EUCAST guidelines. Only 1 center further analyzed the isolates for the presence of KPC by using an immunochromatographic test: KPC K-SeT test (Coris Bioconcept, https://www.corisbio.com). Genetic detection of carbapenemases was performed in only 3 centers by using the Xpert Carba-R assay (Cepheid, http://www.cepheid.com).

Bacterial isolates were subcultured in blood agar medium (Becton Dickinson) and incubated overnight at 37°C. DNA was extracted from a liquid suspension of the isolated colonies by using the Maxwell 16 Cell DNA Purification Kit SEV (Promega, https://www.promega.com) in combination with a Maxwell 16 Instrument (Promega) for automated isolation of genomic DNA. All strains were sequenced with the Illumina NextSeq500 platform (Illumina Inc., htpps://illumina.com), with a paired-end run of 2 by 150 bp, after Nextera XT (Illumina) paired-end library preparation.

Sequencing reads were assembled using SPAdes Genome version 3.13 (http://cab.spbu.ru/software/spades) with accurate de novo settings (*4*). The assembled contigs were evaluated with an automated bioinformatic pipeline for AMR gene detection (ResFinder v.3.0, https://cge.cbs.dtu.dk/services/ResFinder), available at the Center for Genomic Epidemiology (*5*). Phylogenic analysis was based on core-genome single nucleotide polymorphism (SNP) sequences (47,895 SNPs) obtained from the analysis of 989 *Klebsiella pneumoniae* genomes using kSNP3.0 (https://sourceforge.net/projects/ksnp). Parametric maximum-likelihood estimation (model: GTR+G+I) analysis with 1,000 bootstrap estimates was used to infer the phylogeny; IQ-TREE (http://www.iqtree.org) was used to generate the tree; iTOL (https://itol.embl.de) was used for graphic representation of the tree (Figure 2). Major branches have bootstrap values >0.75 for branch support (*6*).

Statistical Analysis

Categorical variables are presented as frequency and proportion (%) and continuous variables as median, lower and upper quartiles (Q1–Q3). The most common reasons for nonenrollment were a delay in communications between the microbiologist and physician responsible for patient enrollment, patient transfer to other facilities before being enrolled, or both.

To estimate the various indicators describing KPC-*Kp* epidemiology that emerged from the implemented surveillance system, without modifying or interfering with current clinical practices and hospital policies, we retrieved information on all hospitalized patients, irrespective of KPC-*Kp* surveillance, available from the hospitals' administrative data repositories for the year 2017. Centers were considered for the calculation of KPC-*Kp* prevalence and cumulative incidence of infection only if they provided the administrative database of all in-patient admissions and enrolled ≥85% of all adult patients for whom a positive KPC-*Kp* isolate was collected, as recorded in the microbiology laboratory database. Among 15 participating centers, 8 were included in our calculations. We excluded 6 centers because their period of enrollment did not cover all 12 months of 2017 (Appendix Figure 1), and we excluded 1 center that had retrieved data for <85% of the KPC-*Kp* patients registered in the microbiology laboratory database during 2017 (Figure 1). We then merged this database with information on KPC-*Kp* patients available in the study cohort database.

KPC-*Kp* patients were classified, according to the most clinically relevant KPC-*Kp* event, into 3 mutually-exclusive groups: never infected-colonized patients (Ncol), patients infected within ≤48 hours since hospital admission (Nadm), and those with an infection occurring later during hospitalization (i.e., hospital-acquired infections, Nstay). The various measures were calculated as follow: the prevalence of KPC-*Kp* in hospitalized patients in the region of Lombardy during 2017 (Ptot = 1,000*(Ncol+Nadm+Nstay)/Ntot), the prevalence of KPC-*Kp* non-infected colonized patients in the same population (Pcol = 1000*Ncol/Ntot), the cumulative incidence of acquired infections among hospitalized patients in the region of Lombardy in 2017 (Pinf = 1000*(Nadm+Nstay)/Ntot), and the cumulative incidence of acquired infections occurring >48 hours of hospital admission among hospitalized patients in the region of Lombardy in 2017 (CI = 1,000* Nstay/Ntot). Prevalence/incidence were reported as crude rates and standardized by age (lower or higher than 66 years, the median of the 170,699 patients admitted) and ward of isolation (ICU, infectious diseases, surgery, oncology/hematology, and other medical wards), according to a direct method ($_{std}P$). The standard population was the overall adult population of patients admitted in 2017 in these 8 centers, excluding day admissions and pediatric admissions. We calculated 95% CI by using Poisson distribution (Appendix Figure 3).

To study the role of KPC-*Kp* infection severity on 15-day mortality rates in KPC-*Kp* patients, we considered the exposure variable of KPC-*Kp* infections as severe, mild, or colonized. We conducted a survival analysis in which the time of KPC-*Kp* isolation was taken as time of origin (i.e., t = 0), and the event was hospital death occurring ≤15 days of KPC-*Kp* isolation, thus we censored hospital stays at 15 days. Colonized patients were selected as the reference category since they represented the best available control group because they were hospitalized during the same time and at the same locations in which the KPC-*Kp*-infected cases arose, and had comparable clinical characteristics (Appendix Table 2). Multivariable Cox proportional hazard frailty models on 15-day hospital mortality rates were used to estimate both crude and adjusted hazard ratios (HRs). To select covariates in the Cox proportional hazard mixed models, we constructed several Cox proportional hazard mixed models (i.e., center was entered as random effect) to identify factors independently associated with 15-day hospital mortality rates. In particular, we included the following: previous colonization (yes/no), previous hospitalization (yes/no); isolation ward ICU (yes/no); antimicrobial therapy in the 30 days before hospitalization (yes/no); major surgery in the 30 days before isolation (yes/no); Charlson Index; underlying conditions, including congestive heart failure (yes/no), peripheral vascular disease (yes/no), cerebrovascular disease (yes/no), chronic lung disease (yes/no), chronic renal failure (yes/no), cancer (yes/no), or diabetes (yes/no); central venous catheter at isolation, urinary catheter at isolation, immunosuppressive therapy (yes/no); carbapenem-resistance mechanism (KPC3 vs KPC2); and major clones ST512 (yes/no) or ST307 (yes/no). Only the number of days between admission and KPC-*Kp* isolation were considered for KPC-*Kp* patients as a covariate in the first models. A center-specific random intercept was also included, to adjust for potential center-specific effects. Hospital stays were censored at 15 days. Thus, factors were entered into the adjusted models on the basis of their univariate relation to outcome ($p<0.20$) for differences in confounding factors between types of KPC-*Kp* patients. All factors were biologically plausible with a sound scientific rationale. However, if the Pearson or Spearman correlation coefficient (according to variables distribution), was >0.30, the variable with the lower p value was retained in the model (for example, when central venous catheter was considered, ICU was excluded as the ward of isolation), or if the Charlson Index was included in the multivariable model, components of this score (such as renal failure) were not included separately. The proportional hazards (PH) assumption was checked by graphical diagnostics based on the scaled Schoenfeld

residuals, entering time-dependent covariates for each covariate, included 1 by 1 in the model, and verifying estimated coefficients with $p<0.05$.

When the main targeted therapeutic regimens were compared between the severe and mild infection groups, we used χ^2 and Fisher exact tests to compare categorical variables and *t*-test or Mann-Whitney U test to compare continuous variables. We performed all analyses by using SAS 9.4 software (SAS Institute, Inc., https://www.sas.com) and considered $p\leq0.05$ statistically significant.

References

1. Friedman ND, Kaye KS, Stout JE, McGarry SA, Trivette SL, Briggs JP, et al. Health care—associated bloodstream infections in adults: a reason to change the accepted definition of community-acquired infections. Ann Intern Med. 2002;137:791–7. PubMed https://doi.org/10.7326/0003-4819-137-10-200211190-00007

2. Centers for Disease Control and Prevention. CDC/NHSN surveillance definitions for specific types of infections. Atlanta: the Centers; 2014 [cited 2020 Dec 19]. http://www.socinorte.com/wp-content/uploads/2014/06/17pscNosInfDef_current.pdf

3. European Committee on Antimicrobial Susceptibility Testing (EUCAST). EUCAST guidelines for detection of resistance mechanisms and specific resistances of clinical and/or epidemiological importance. Version 2.0; July 2017 [cited 2020 Dec 19]. https://eucast.org/resistance_mechanisms

4. Nurk S, Bankevich A, Antipov D, Gurevich AA, Korobeynikov A, Lapidus A, et al. Assembling single-cell genomes and mini-metagenomes from chimeric MDA products. J Comput Biol. 2013;20:714–37. PubMed https://doi.org/10.1089/cmb.2013.0084

5. Clausen PT, Zankari E, Aarestrup FM, Lund O. Benchmarking of methods for identification of antimicrobial resistance genes in bacterial whole genome data. J Antimicrob Chemother. 2016;71:2484–8. PubMed https://doi.org/10.1093/jac/dkw184

6. Shakya M, Ahmed SA, Davenport KW, Flynn MC, Lo C-C, Chain PSG. Standardized phylogenetic and molecular evolutionary analysis applied to species across the microbial tree of life. Sci Rep. 2020;10:1723. PubMed https://doi.org/10.1038/s41598-020-58356-1

Appendix Table 1. Description of the 15 study centers and their surveillance protocols (KPC-*Kp* study network)

Center characteristics				Surveillance protocols									Risk factors†		
				Unit, rectal swab or other cultures done weekly									Previous		
Center; no. adm/y	Ter	Teach	No. ICU beds	ICU	TP	OH	Surg	ID	GM	GE	Neph	Ger	Hospitalization	Colonization	Di
G; 51,000	Y	Y	32	Y	N	Y	N	N	N	NA	NA	NA	N	N	N
I; 44,900	Y	Y	24	Y	N	Y	Pancreatic	NA	N	N	N	NA	Y	Y	Y
N; 41,292	N	Y	26	Y	N	Y	N	N	N	N	N	NA	N	N	N
H; 40,000	Y	Y	32	Y	N	N	N	N	N	N	N	N	Y	Y	N
B; 37,000	Y	Y	76	Y	Y	Y	N	N	Y	Y	NA	NA	Y	Y	N
K; 34,908	Y	Y	21	Y	N	N	N	NA	N	N	NA	N	N	Y	Y
E; 31,297	Y	Y	42	Y	Y	N	Abd/hep	N	N	N	NA	NA	Y	Y	N
A; 27,600	Y	Y	24	Y	NA	Y	N	N	N	N	N	N	Y	Y	Y
D; 27,000	Y	N	30	Y	NA	N	N	N	N	NA	N	NA	Y	Y	Y
M; 21,480	N	N	8	Y	NA	Y	N	N	N	NA	N	NA	Y	Y	N
O; 19,225	Y	Y	17	Y	N	N	N	Y	N	NA	N	NA	Y	Y	Y
L; 18,287	Y	Y	6	Y	NA	NA	N	N	N	N	N	NA	N	N	Y
C; 18,167	N	N	8	Y	NA	Y	Urology	Y	N	N	Y	NA	N	Y	Y
P; 18,000	N	N	7	Y	NA	NA	N	N	N	NA	N	Y	Y	Y	Y
F; 15,869	Y	Y	14	Y	NA	NA	Abd	Y	Y	N	Y	NA	N	N	N

*Abd, abdominal; adm, admissions; Di, dialysis; GE, gastroenterology; Ger, gerontology; GM, general medical; hep, hepatic; ICU, intensive care unit; ID, infectious diseases; NA, not applicable; Neph, nephrology; OH, onco-hematology; Surg, surgical; Teach, teaching; Ter, tertiary; TP, solid organ transplant.
†Risk factors considered in active surveillance at admission.

Appendix Table 2. Characteristics and severity of KPC-*Kp* among patient enrolled from 15 healthcare centers during 2016–2018, Italy*

Characteristics	Severity of KPC-*Kp* infection: Severe	Mild	Colonized
All	221 (100)	109 (100)	741 (100)
Sex			
M	149 (67.4)	71 (65.1)	474 (64.0)
F	72 (32.5)	38 (34.9)	267 (36.0)
Median age (IQR)	70 (60–78)	75 (64–82)	72 (61–80)
Healthcare exposures before KPC-*Kp* onset			
Previous KPC-*Kp* colonization in the past 12 mo.	102 (46.2)	46 (42.2)	185 (25.0)
Previous hospitalization in the past 12 mo.	168 (76.0)	85 (78.0)	612 (82.6)
Antimicrobial therapy in the 30 d before hospitalization	166 (75.1)	85 (78.0)	531 (71.7)
Major surgery in the past 30 d	68 (30.8)	32 (29.4)	162 (21.9)
Underlying conditions†			
All underlying conditions	206 (93.2)	103 (94.5)	680 (91.8)
Congestive heart failure	35 (15.8)	20 (18.4)	137 (18.5)
Peripheral vascular disease	47 (21.3)	21 (19.3)	129 (17.4)
Cerebrovascular disease	35 (15.8)	19 (17.4)	151 (20.4)
Chronic lung disease	30 (13.6)	24 (22.0)	148 (20.0)
Chronic renal failure	57 (25.8)	34 (31.2)	213 (28.7)
Cancer	54 (24.4)	35 (32.1)	155 (20.9)
Diabetes	25 (11.3)	24 (22.0)	114 (15.4)
Any combination of CHF, PVD, or CRF	90 (40.7)	46 (42.2)	321 (43.3)
Other markers of the severity of underlying illness			
Median Charlson Index (IQR)	5.0 (3.0–7.0)	6.0 (4.0–9.0)	6.0 (4.0–8.0)
Central venous catheter at isolation	118 (53.4)	39 (35.8)	257 (34.7)
Urinary catheter at isolation	120 (54.3)	60 (55.1)	382 (51.5)
Immunosuppressive therapy	55 (24.9)	20 (18.4)	134 (18.1)

*Data are no. (%) except where otherwise noted. Severe infection included bloodstream or lower respiratory tract infection plus septic shock from other sites; Mild infection included infections from other sites; and colonizedsur patients were identified through surveillance protocols. CHF, congestive heart failure; CRF, chronic renal failure; KPC-*Kp*, *Klebsiella pneumoniae* carbapenemase-producing *Klebsiella pneumoniae*; PVD, peripheral vascular disease.
†Underlying conditions were included when present in ≥10% of all patients.

Appendix Table 3. Number of isolates according to sequence type and *Klebsiella pneumoniae* carbapenemase-producing variants reported for 15 healthcare centers participating in KPC-*Kp* surveillance, Italy*

Center code; total no. isolates	No. KPC variants		Sequence types, no.				
	KPC-2	KPC-3	ST101	ST258	ST307	ST512	Others
All	313	676	57	71	326	441	94
A; n = 234	58	176	6	11	79	118	20
B; n = 84	36	48	3	7	26	43	5
C; n = 50	6	44	3	3	2	34	8
D; n = 50	18	32	1	2	20	22	5
E; n = 169	80	89	6	15	73	49	26
F; n = 21	9	12	0	4	8	8	1
G; n = 72	10	62	5	4	25	34	4
H; n = 78	13	65	11	2	10	48	7
I; n = 76	14	62	7	6	8	44	11
K; n = 71	21	50	6	3	41	19	2
L; n = 51	35	16	8	12	26	5	0
M; n = 7	1	6	0	0	0	3	4
N; n = 8	1	7	0	0	1	7	0
O; n = 12	7	5	0	0	7	5	0
P; n = 6	4	2	1	2	0	2	1

*KPC-*Kp*, *Klebsiella pneumoniae* carbapenemase-producing *Klebsiella pneumoniae*; ST, sequence type

Appendix Table 4. Overall in-hospital mortality rates among patients infected or colonized by KPC-*Kp* according to selected characteristics*

Characteristics	KPC-*Kp* colonized, n = 741		KPC-*Kp* infected, n = 330	
	**No. (%)**	**95% CI**	**No. (%)**	**95% CI**
Overall rates	152 (20.5)	17.7%–27.6%	113 (34.2)	29.2%–39.6%
Median days from hospitalization to strain isolation (IQR)	7 (1.0–17.5)	NA	29 (15.0–47.0)	NA
Median days from strain isolation to death (IQR)	8 (3.0–17.5)	NA	12 (5.0–18.0)	NA
Intensive care unit admission				
Y	70/192 (36.5)	29.6%–43.7%	39/75 (52.0)	40.2%–63.7%
N	82/549 (14.9)	12.1%–18.2%	74/255 (29.0)	23.5%–35.0%
Previous KPC-*Kp* colonization	17/185 (9.2)	5.4%–14.3%	55/148 (37.2)	29.4%–45.5%
Previous hospitalization	118/612 (19.3)	16.2%–22.6%	85/253 (33.6)	27.8%–39.8%
Antimicrobial therapy in the 30 d before hospitalization	112/531 (21.1)	17.7%–24.8%	89/251 (35.5)	29.5%–41.7%
Major surgery	36/162 (22.2)	16.1%–29.4%	40/100 (40.0)	30.3%–50.3%
Underlying conditions	145/680 (21.3)	18.3%–24.6%	109/309 (35.3)	29.9%–40.9%
Carbapenem-resistance mechanism†				
KPC-2	35/219 (16.0)	11.4%–21.5%	36/94 (38.3)	28.5%–48.9%
KPC-3	96/440 (21.8)	18.0%–26.0%	77/236 (32.6)	26.7%–39.0%
Most frequent clones				
ST101	10/40 (25.0)	12.7%–41.2%	7/17 (41.2)	18.4%–67.1%
ST258	8/49 (16.3)	7.3%–29.7%	7/22 (31.8)	13.9%–54.9%
ST307	42/220 (19.1)	14.1%–24.9%	34/106 (32.1)	23.3%–41.8%
ST512	60/283 (21.2)	16.6%–26.4%	56/158 (35.4)	28.0%–43.4%
Infection severity‡				
Mild	NA	NA	23/109 (21.1)	13.9%–30.0%
Severe	NA	NA	90/221 (40.7)	34.2%–47.5%
Bloodstream infections	NA	NA	65/179 (36.3)	25.1%–39.2%
With septic-shock	NA	NA	24/45 (53.3)	37.9%–68.3%
Without septic-shock	NA	NA	41/134 (30.6)	22.9%–39.1%

*Among colonized patients, 82 had no available data on genotyping. KPC-*Kp*, *Klebsiella pneumoniae* carbapenemase-producing *Klebsiella pneumoniae*; ST, sequence type.
‡Severe infection included bloodstream or lower respiratory tract infection plus septic shock from other sites; Mild infection included infections from other sites; and colonizedsur patients were identified through surveillance protocols.

Appendix Table 5. Relations between empirical antimicrobial drug appropriateness and selected characteristics among patients with KPC-*Kp* infections, Italy*

	Empirical therapy			
Characteristics	Inadequate	Adequate	χ^2 p value	p value†
All	138 (100)	159 (100)	NA	NA
Median age (IQR)	74 (60–80)	68 (73–78)	0.260	0.757
Charlson Index	6 (4–8)	5 (4–8)	0.365	0.984
Intensive care unit admission				
Y	31 (47.7)	34 (52.3)	0.822	0.320
Previous KPC-*Kp* colonization during the current hospitalization				
Y	21 (33.9)	41 (66.1)	0.025	0.056
N	117 (49.8)	118 (50.2)	Referent	Referent
Previous KPC-*Kp* colonization in the past 12 mo.				
Y	44 (32.6)	91 (67.4)	<0.001	<0.001
N	93 (57.8)	68 (42.2)	Referent	Referent
Previous hospitalization in the past 12 mo.				
Y	107 (46.3)	124 (53.7)	0.981	0.943
N	30 (46.2)	35 (53.8)	Referent	Referent
Antimicrobial therapy in the 30 d before hospitalization				
Y	112 (48.9)	117 (51.1)	0.094	0.062
N	25 (37.3)	42 (62.7)	Referent	Referent
Major surgery				
Y	50 (56.2)	39 (43.8)	0.028	0.016
N	88 (42.3)	120 (57.7)	Referent	Referent
KPC-*Kp* infection severity‡				
Severe	74 (38.1)	120 (61.9)	<0.001	0.0003
Mild	64 (62.1)	39 (37.9)	Referent	Referent

*Antimicrobial drug appropriateness determined according to the bacteria resistance profile and selected characteristics. Thirty-three patients were excluded: 17 had follow-up <3 d, 16 had no data on empirical therapies. Values represent no. (%) except where otherwise indicated. KPC-*Kp*, *Klebsiella pneumoniae* carbapenemase-producing *Klebsiella pneumoniae*.
†Estimates from multivariable mixed logistic model adjusted by center (as random effect), age and type of KPC-*Kp* infection
‡Severe infection included bloodstream or lower respiratory tract infection plus septic shock from other sites; mild infection included infections from other sites.

Appendix Table 6. Main targeted therapeutic regimens according to the severity of KPC-*Kp* infection, 2016–2018, Italy*

Therapy regimens	All	Severe	Mild	p value†
All infections	297 (100)	194 (100)	104 (100)	NA
Targeted therapy				
All therapies‡	282 (94.9)	182 (86.4)	100 (94.9)	NA
Double carbapenem	69 (24.5)	49 (26.9)	20 (20.0)	0.196
Colistin + tigecycline + carbapenem	53 (18.8)	45 (24.7)	8 (8.0)	0.001
Gentamicin + tigecycline + carbapenem	15 (5.3)	11 (6.0)	4 (4.0)	0.585
Colistin + carbapenem	29 (10.3)	19 (10.4)	10 (10.0)	0.907
Gentamicin + carbapenem	14 (5.0)	4 (2.2)	10 (10.0)	0.007
Fosfomycin + carbapenem	10 (3.5)	5 (2.8)	5 (5.0)	0.333
Tigecycline + carbapenem	9 (3.2)	7 (3.9)	2 (2.0)	0.499
Gentamicin monotherapy	10 (3.5)	1 (0.5)	9 (9.0)	0.001
Fosfomycin monotherapy	10 (3.5)	2 (1.1)	8 (8.0)	0.005
Tigecycline monotherapy	9 (3.2)	5 (2.7)	4 (4.0)	0.725
CAZ-AVI combined with others, n = 39§	26 (66.7)	19/24 (79.2)	7/15 (46.7)	0.033

*CAZ-AVI, ceftazidime-avibactam; KPC-*Kp*, *Klebsiella pneumoniae* carbapenemase-producing *Klebsiella pneumoniae*.
†p values refer to χ^2 or Fisher exact test, when appropriate.
‡Fifteen patients had missing details of therapies.
§CAZ-AVI became available in February 2018; only 39 of 295 patients who received a targeted therapy were enrolled after that date: 24 had bloodstream or lower respiratory tract infections and 15 had infections from other sites. An additional 2 patients received CAZ-AVI before February 2018 for compassionate use. All patient infections had susceptibility to CAZ-AVI.

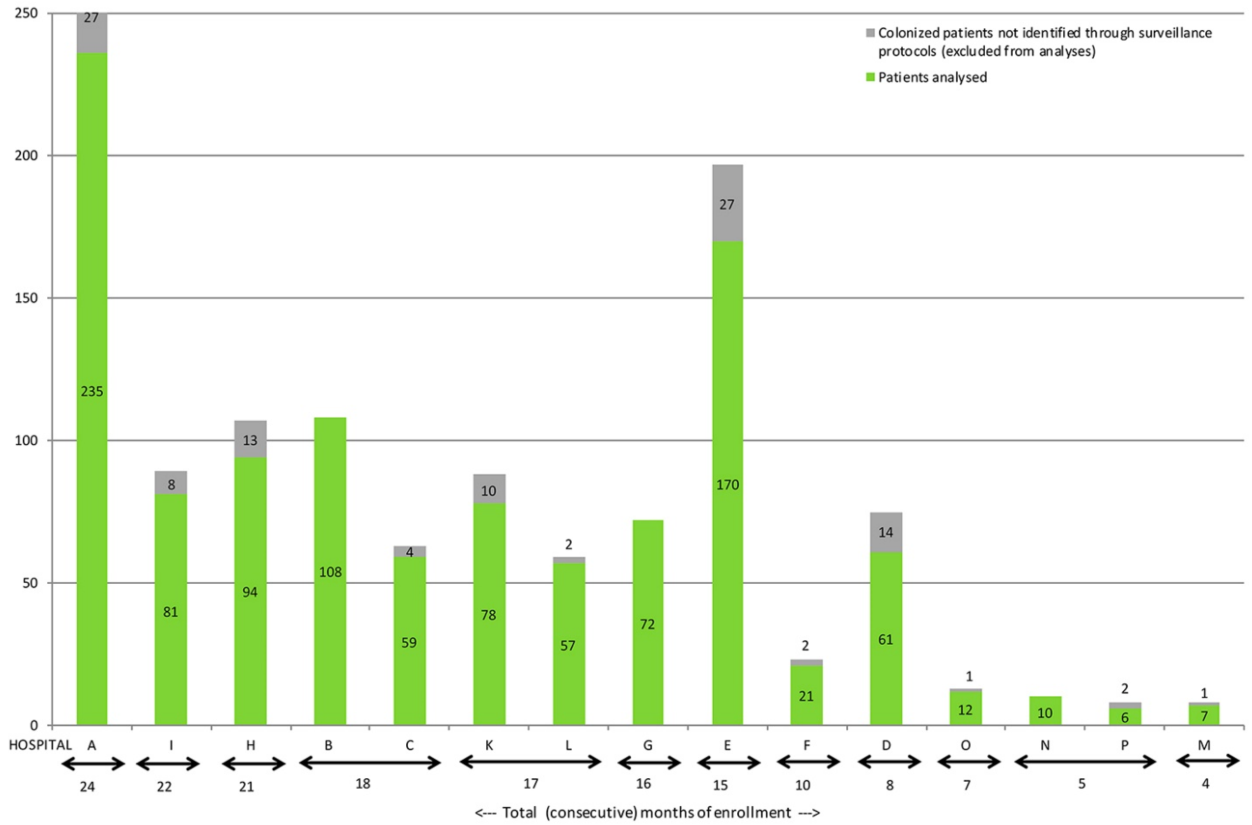

Appendix Figure 1. Number of patients and months of recruitment among participating hospitals in a study of *Klebsiella pneumoniae* carbapenemase-producing *Klebsiella pneumoniae*, Italy. Centers A–P are shown in decreasing order of the number of months of consecutive participation.

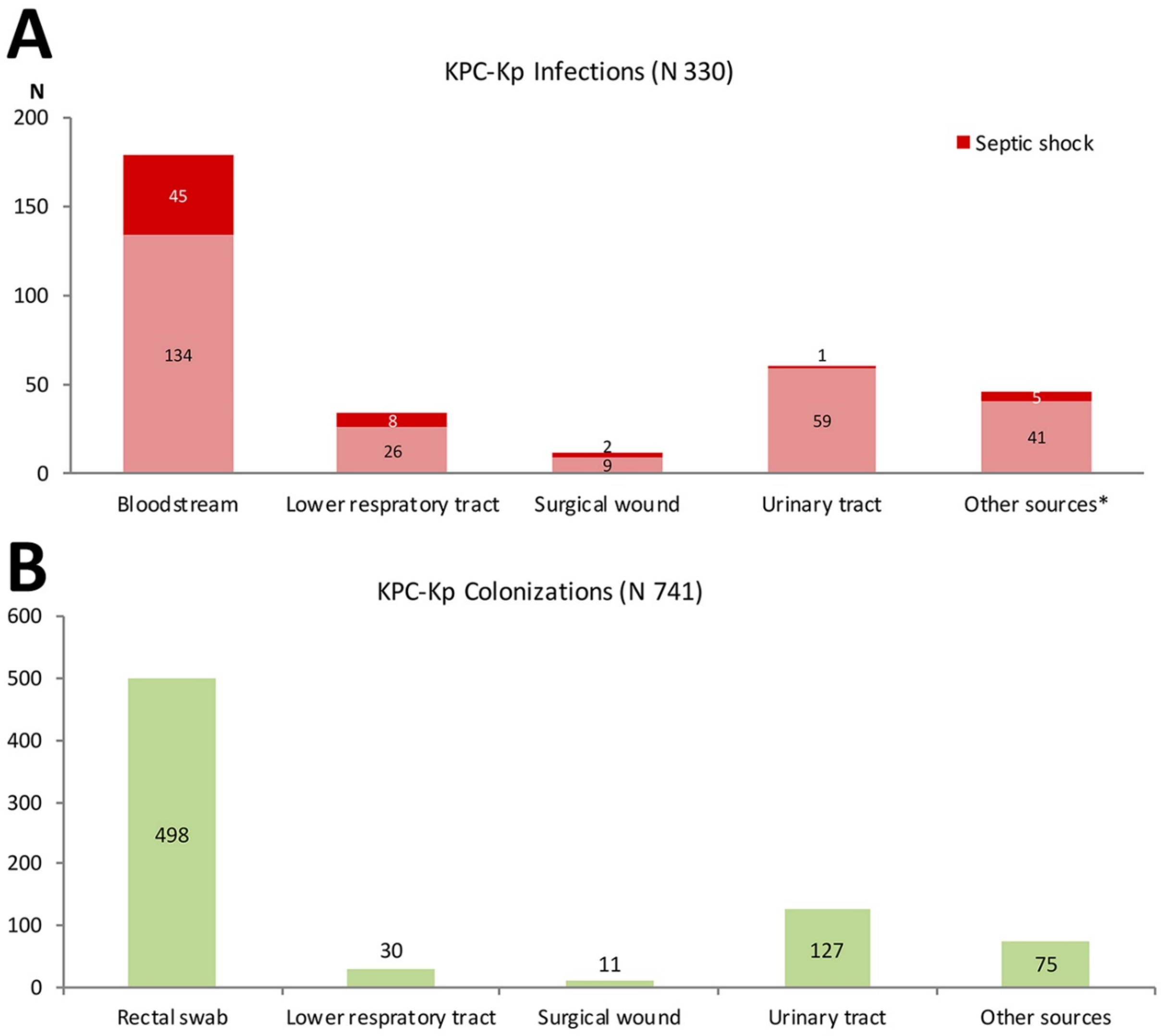

Appendix Figure 2. Source of isolation for the *Klebsiella pneumoniae* carbapenemase-producing *Klebsiella pneumoniae* (KPC-*Kp*) infection and colonization, Italy. Isolations sources for 330 patients with KPC-*Kp* infection (A) and 741 colonized patients (B). *Other sources include 13 from pus and 33 from other sources, reported for <4 patients.

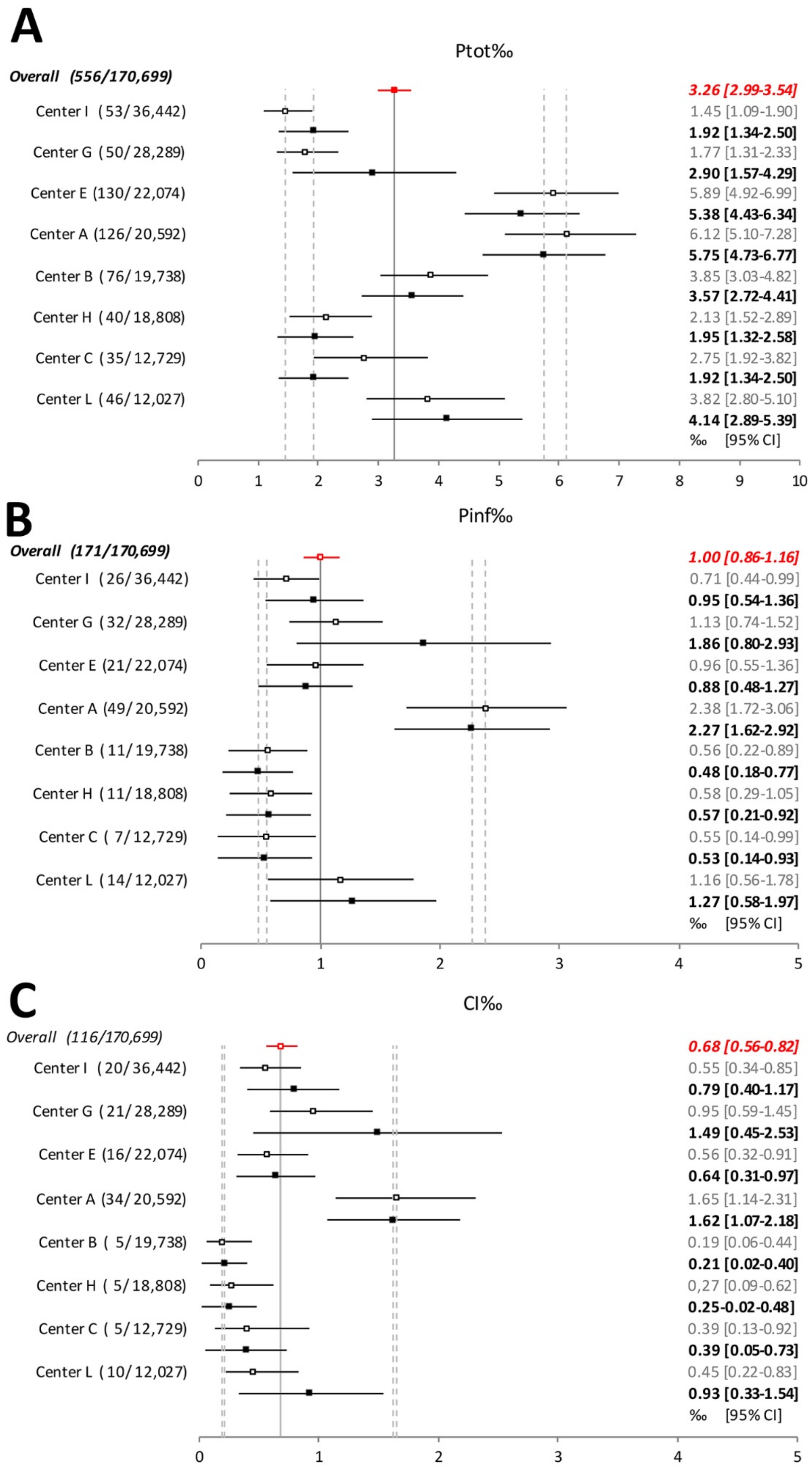
A
Ptot‰
Overall (556/170,699)
3.26 [2.99-3.54]
Center I (53/36,442)
1.45 [1.09-1.90]
1.92 [1.34-2.50]
Center G (50/28,289)
1.77 [1.31-2.33]
2.90 [1.57-4.29]
Center E (130/22,074)
5.89 [4.92-6.99]
5.38 [4.43-6.34]
Center A (126/20,592)
6.12 [5.10-7.28]
5.75 [4.73-6.77]
Center B (76/19,738)
3.85 [3.03-4.82]
3.57 [2.72-4.41]
Center H (40/18,808)
2.13 [1.52-2.89]
1.95 [1.32-2.58]
Center C (35/12,729)
2.75 [1.92-3.82]
1.92 [1.34-2.50]
Center L (46/12,027)
3.82 [2.80-5.10]
4.14 [2.89-5.39]
‰ [95% CI]
0 1 2 3 4 5 6 7 8 9 10
B
Pinf‰
Overall (171/170,699)
1.00 [0.86-1.16]
Center I (26/36,442)
0.71 [0.44-0.99]
0.95 [0.54-1.36]
Center G (32/28,289)
1.13 [0.74-1.52]
1.86 [0.80-2.93]
Center E (21/22,074)
0.96 [0.55-1.36]
0.88 [0.48-1.27]
Center A (49/20,592)
2.38 [1.72-3.06]
2.27 [1.62-2.92]
Center B (11/19,738)
0.56 [0.22-0.89]
0.48 [0.18-0.77]
Center H (11/18,808)
0.58 [0.29-1.05]
0.57 [0.21-0.92]
Center C (7/12,729)
0.55 [0.14-0.99]
0.53 [0.14-0.93]
Center L (14/12,027)
1.16 [0.56-1.78]
1.27 [0.58-1.97]
‰ [95% CI]
0 1 2 3 4 5
C
CI‰
Overall (116/170,699)
0.68 [0.56-0.82]
Center I (20/36,442)
0.55 [0.34-0.85]
0.79 [0.40-1.17]
Center G (21/28,289)
0.95 [0.59-1.45]
1.49 [0.45-2.53]
Center E (16/22,074)
0.56 [0.32-0.91]
0.64 [0.31-0.97]
Center A (34/20,592)
1.65 [1.14-2.31]
1.62 [1.07-2.18]
Center B (5/19,738)
0.19 [0.06-0.44]
0.21 [0.02-0.40]
Center H (5/18,808)
0,27 [0.09-0.62]
0.25-0.02-0.48]
Center C (5/12,729)
0.39 [0.13-0.92]
0.39 [0.05-0.73]
Center L (10/12,027)
0.45 [0.22-0.83]
0.93 [0.33-1.54]
‰ [95% CI]
0 1 2 3 4 5

Appendix Figure 3. The number of *Klebsiella pneumoniae* carbapenemase-producing *Klebsiella pneumoniae* (KPC-*Kp*) isolates per number of hospital admissions among 8 healthcare centers, Italy, 2017. Centers are listed in decreasing order of number of admissions. Right column represent percent [95% CI]; red text indicates value for all centers combined; bold text indicates values standardized by age and ward of isolation. A) Prevalence of KPC-*Kp* per per 1,000 admissions (Ptot‰); B) cumulative incidence of acquired KPC-*Kp* infections among hospitalized patients (Pinf‰); and C) cumulative incidence of acquired KPC-*Kp* infections occurring >48 hours of hospital admission among hospitalized patients (CI‰). Bars show median (squares) and 95% CI for each center. Empty squares identify crude and black squares standardized data by age and ward of isolation. Vertical solid lines represent the value of prevalence/cumulative incidence obtained for all centers combined; vertical dotted lines represent the lowest and highest values obtained for the estimates of 95% confidence intervals.
